# Concerted genetic and transcriptomic shifts underlie adaptation to a latitudinal thermal gradient within a widespread mantis shrimp

**DOI:** 10.1186/s12862-025-02452-1

**Published:** 2025-10-17

**Authors:** Liwen Zhang, Zhongli Sha, Jiao Cheng

**Affiliations:** 1 Department of Marine Organism Taxonomy and Phylogeny, Institute of Oceanology, Chinese Academy of Sciences, Qingdao, 266071 China; 2 Laboratory for Marine Biology and Biotechnology, Qingdao Marine Science and Technology Center, Qingdao, 266237 China; 3 University of Chinese Academy of Sciences, Beijingddd, 100049 China

**Keywords:** Comparative population transcriptomics, Candidate gene approach, Gene expression, Genetic variation, Thermal adaptation

## Abstract

**Background:**

Widely dispersed species are often found across heterogeneous environments, which can result in localized adaptive divergence among populations. While previous studies have highlighted the role of gene sequence variation in shaping adaptive divergence patterns, the contribution of gene expression changes remains poorly characterized. We explore this in a widespread mantis shrimp (*Oratosquilla oratoria*), distributed along the well-defined thermal clines in the Northwestern Pacific (NWP), to dissect the interplay between sequence and expression variation in adaptation to a latitudinal thermal gradient.

**Results:**

Population transcriptomics of 51 *O. oratoria* individuals from four populations along the NWP latitudinal gradient revealed a significant north–south population structure at both the sequence and expression levels. The absence of isolation by distance underscored the role of natural selection. Positive correlations between nucleotide diversity and expression diversity within and among populations suggest that genetic and expression variation collaboratively enhance *O. oratoria*’ s survival in diverse habitats. By integrating with knowledge of gene functions from a reverse ecology perspective, we identified an over-representation of temperature-relevant candidate gene transcripts (CGTs) contributing significantly to the expression divergence among *O. oratoria* populations, whereas no such over-representation was observed in highly divergent CGTs across different latitudinal populations. Compared to the gene set, the differentially expressed and highly divergent CGTs exhibit greater overlap in functional categories, including the biological process and molecular function GO terms.

**Conclusions:**

Our findings demonstrate that local thermal selection may have acted on gene expression levels, thereby prompting further investigation into potential non-coding regulatory changes. Additionally, the functional consistency of differentially-expressed and highly divergent CGTs compared to a shared gene set implies alternative ways for *O. oratoria* to respond to thermally environmental stresses across latitudes. This work provides evidence of how gene sequence and expression changes work in concert in a widespread species in response to a highly selective environment.

**Supplementary Information:**

The online version contains supplementary material available at 10.1186/s12862-025-02452-1.

## Background

Understanding the mechanisms by which species persist in diverse and changing environments remains one of the fundamental questions in evolutionary biology [1]. Widely distributed species generally consist of populations inhabiting heterogeneous environments. Such environmental heterogeneity throughout a species range can impose spatially varying selection on local populations despite the absence of geographic isolation [2, 3]. Comparisons among populations distributed along environmental gradients provide important insights into how species’ genomes diverge in response to disparate environmental pressures [4, 5]. However, questions have remained as to pinpoint the genetic underpinnings of local adaptation, especially of locally adapted traits, in natural populations inhabiting heterogeneous environments. The polygenic basis of physiological tolerance traits further complicates the link between genetic variation and ecological phenotypes or adaptation [6]. Moreover, challenges in constructing multi-generational pedigrees hinder the application of quantitative genetic approaches to assess heritable variation in traits and to test hypotheses regarding the molecular mechanisms underlying adaptation [7].

A promising approach to disentangle this complexity is through integrating gene function and molecular evolutionary approaches that can enhance our understanding of how genomic changes affect the adaptation of organisms to their environments [8]. On the one hand, molecular evolutionary approaches enable the study of how natural selection directly alters gene sequences, even without prior knowledge of the specific phenotypic changes during adaptation [9]. On the other hand, known classifications of gene function can help to understand how molecular traits influence physiological differences between species or populations, even when these responses are controlled by many interrelated genes [10]. In addition to gene sequence changes, alterations in gene expression may also drive adaptive evolution via linking heritable molecular changes at the DNA level with fitness-related phenotypic traits [11, 12]. Nowadays, a central objective in current molecular evolutionary studies should involve deciphering the origins of gene expression and sequence diversity, along with their adaptive consequences, thereby advancing mechanistic understanding of biological adaptability at both the genetic and expression levels [13].

**Fig. 1:**
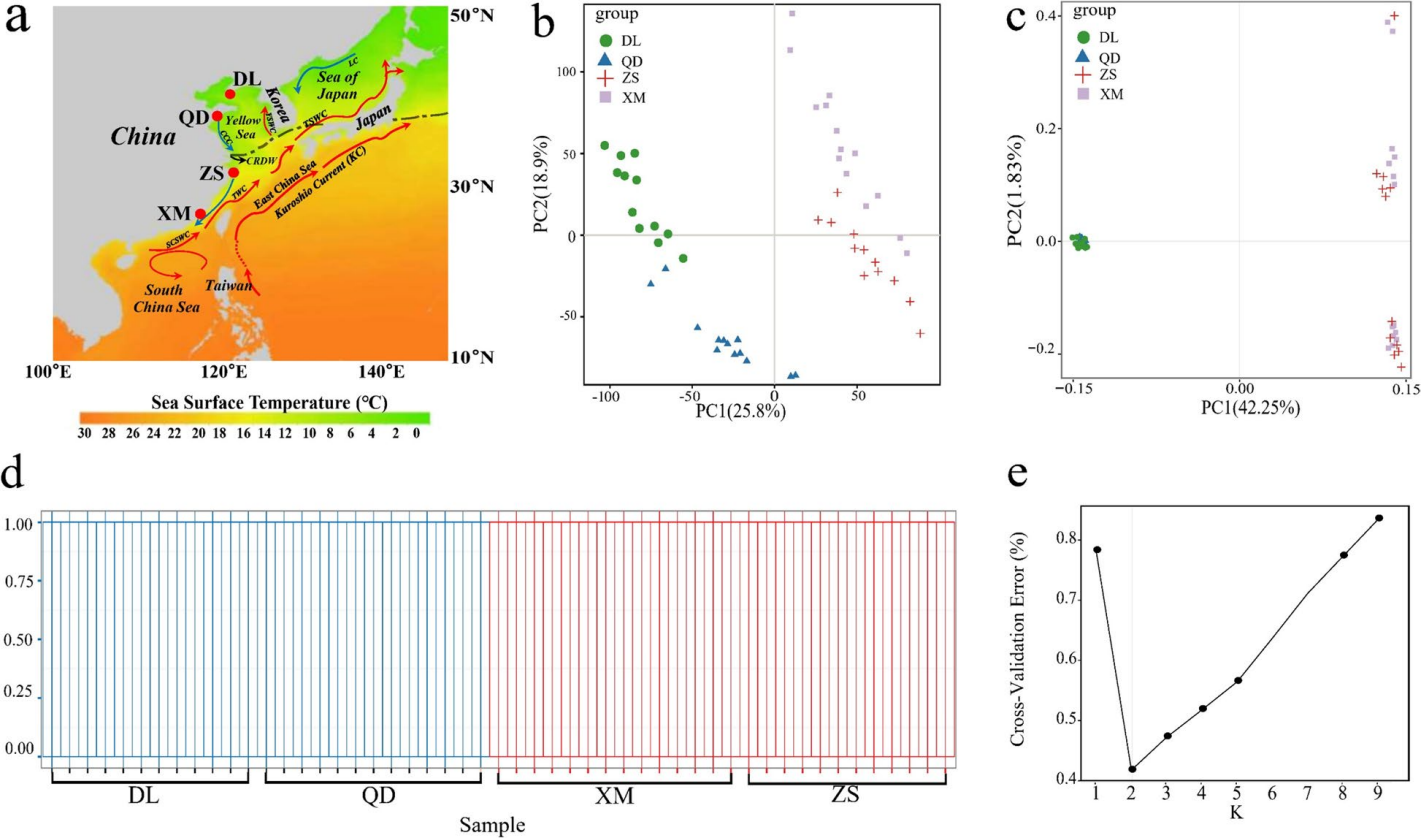
Sampling locations and population genetic structure in *O. oratoria* based on expression and SNP data. **a** Sampling locations. The map also shows Sea Surface Temperature (SST) in the NWP during winter (2010–2020) (https://bio-oracle.org). SCSWC, South China Sea Warm Current; TWC, Taiwan Warm Current; CCC, China Coastal Current; CRDW, Changjiang River Diluted Water; YSWC, Yellow Sea Warm Current; TSWC, Tsushima Warm Current; LC, Lima Current. **b** Population genetic analyses using PCA based on FPKM. **b** PCA based on SNPs. **d** Population structure plots with K = 2. **e** Plot of CV error

The Northwestern Pacific (NWP) coastline comprises two ecologically distinct marine bioregions that are structed along a thermal gradient. A biogeographic boundary extending from the Yangtze River Estuary in China northeastward through Jeju Island (Korea) to southern Japan (Fig. 1a) delineates the cold-temperate North Pacific Biotic Province from the tropical-subtropical Indo-West Pacific Biotic Province [14]. This provides a unique opportunity to investigate how latitudinal thermal gradient in species diversification are formed, as temperature affects organisms through multiple levels of biological organization from molecular biochemistry to organismal physiology [15, 16]. The Japanese mantis shrimp, *Oratosquilla oratoria* (De Haan, 1844), is widely distributed along the NWP coastline that comprise cryptic species whose ranges are limited by the boundaries between bioregions [17–19], and which exhibited divergent transcriptomic signatures in response to heat stress [20]. As such, *O. oratoria* may serve as a key model to delineate the effects of temperature-mediated adaptation on marine diversification. Nevertheless, we know little about the mechanisms underlying thermal adaptation in this widespread mantis shrimp, as well as the relative contribution of genetic and expression variation in its local adaptation to thermally environmental gradient.

Here, we employed population transcriptomics to simultaneously estimate expression and sequence divergence among four natural populations of *O. oratoria* along the NWP latitudinal gradient. Given the scarcity of information on the relationship between gene and phenotype in non-model organisms, we therefore utilize a reverse ecology approach that allows for assessing the importance of environmental factors in shaping the divergence among natural populations [21]. To do this, a set of temperature-relevant candidate genes that are previously assumed to contribute to temperature adaptation was first constructed from a broad variety of studies on arthropods. Then, we seek to unravel the genetic mechanisms driving adaptive divergence in *O. oratoria* populations under temperature-mediated selection by examining population-level gene expression and sequence variation in transcripts orthologous to temperature-relevant candidate genes. Our study aimed to investigate three key aspects: (1) the influence of genetic and expression diversity on the environmental adaptation pattern of *O. oratoria*, (2) the contribution of genetic and expression variation to thermally adaptive divergence among *O. oratoria* populations, and (3) the functional interplay between genetic and expression variation in the temperature adaptation of *O. oratoria*. Such a systematic integration of expression polymorphisms and sequence variants in the context of known physiological adaptations enables us to better understand the genetic underpinnings of local adaptation in natural populations inhabiting heterogeneous environments.

## Materials and methods

### Sample collection, RNA extraction and RNA-Seq

To investigate the transcriptomic characteristics of *O. oratoria* populations, we collected 51 healthy, adult, male *O. oratoria* individuals from four locations along the Chinese coast (DL-Dalian, *n* = 12; QD-Qingdao, *n* = 13; ZS-Zhoushan, *n* = 12; XM-Xiamen, *n* = 14) (Fig. 1a), where the annual average temperature gradually increases and the annual temperature fluctuation range gradually decreases from north to south [22]. The sample collection was completed from May to July 2019, i.e. from the end of spring to the beginning of summer, and no extreme weather events occurred during this period. Muscle tissue in marine organisms is a key energy and amino acid reservoir and shows significant responses to temperature changes. Moreover, muscle can undergo remodeling as a response to temperature stress, which helps the organism adapt to movement and load [20]. Therefore, the abdominal muscle of *O. oratoria* individuals was collected immediately in the field and stored at liquid nitrogen for storage until RNA extraction.

Total RNA of abdominal muscle was extracted using TRIzol Reagent kit (Invitrogen, Carlsbad, CA, USA) according to the manufacturer*’* s instructions. The quality of the isolated RNA was assessed using an Agilent 2100 Bioanalyzer (Agilent Technologies, Palo Alto, CA, USA) and verified by RNase free agarose gel electrophoresis. Total RNA was extracted and mRNA enriched using Oligo(dT) beads. The mRNA was then fragmented and reverse transcribed into cDNA with random primers. Second-strand cDNA synthesis was performed with DNA polymerase I, RNase H, and dNTPs. The cDNA was purified with QiaQuick PCR extraction kit (Qiagen, Venlo, The Netherlands), end-repaired, poly(A)-tailed, and Illumina adapters were ligated. Products were size-selected by agarose gel electrophoresis, amplified, and sequenced on the Illumina HiSeq™ 4000. The generated raw reads were then filtered and trimmed for quality control using fastp software [23], resulting in clean reads for downstream analyses.

### Genetic variation and population structure based on SNP data

Clean reads from each individual were aligned to the reference *O. oratoria* SMRT full-length transcriptome [24], consisting of 42,735 unigenes with an N50 length of 3,472 bp, using bowtie2 v2.2.5 [25] with default settings. The mapped reads were then sorted and indexed with SAMtools v1.19 [26], and candidate SNPs were then identified with BCFtools v1.9. To increase the accuracy of SNP detection, VCFtools v0.1.16 [27] was applied to filter for biallelic SNPs and to exclude those with > 50% missing data, average depth < 6, minor allele frequency (MAF) < 0.02 and quality score ≤ 10. Ultimately, 264,355 high-quality SNPs were identified and used for subsequent analysis.

Using the high-quality SNPs, nucleotide diversity (π) per unigene and pairwise genetic differentiation (*F*_ST_) between populations were calculated with VCFtools v0.1.16. The population genetic structure of *O. oratoria* based on the SNP data was analyzed through principal component analysis (PCA) with GCTA v1.93.2 [28] and Bayesian clustering with a set of predefined genetic clusters values (K = 2–10) using Admixture v 1.3.0 [29]. The optimal number of K was determined by evaluating the cross-validation error.

### Variation in gene expression and population differentiation based on FPKM

All Illumina clean reads were aligned to reference transcriptome of *O. oratoria* using bowtie2 v2.2.5 [25], and transcript expression was quantified in terms of the expected fragments per kilobase of transcript per million fragments (FPKM) using RSEM software with default settings [30]. Reads with low or highly variable expression (library average < 4 or standard deviation over all libraries > library average in each population) were removed to mitigate sequencing errors and the effect of outlier individuals on statistical comparisons [31]. After filtering, 14,291 unigenes were retained.

Population gene expression (Ep) was calculated as the mean FPKM of sampled individuals ($\text{Ep}=\frac{{\sum }_{\text{i}=1}^{\text{n}}\text{Ei}}{\text{n}}$, where n is the number of individuals sampled from the population and Ei is the FPKM of a given gene of the ith individual in the population), and the gene expression diversity (Ed) of each *O. oratoria* population was measured as the deviation from the mean expression ($\text{Ed}=\frac{{\sum }_{\text{i}=1}^{\text{n}}|\text{Ei}-\text{Ep}|}{(\text{n}-1)\text{Ep}}$). Both calculations followed the method of Xu et al. [32]. For the 51 individuals, relationships were analyzed by PCA based on normalized FPKM values using the R package MISSMDA [33]. The gene expression relationship among populations (Ep similarity) was characterized by Pearson correlation coefficients (*r*), which were calculated based on the average correlation coefficients of Ep distribution of individuals.

### Assessment of patterns of population expression and genetic diversity

Mantel tests in the R package vegan were used to assess the relationships between geographic, genetic (*F*_ST_) and expression (Ep) distances, with 1,000 permutations [34], while geographic distances between populations were calculated using the R package geosphere [35]. The correlation between genetic diversity and expression diversity was investigated by calculating correlation coefficients for Ed and π of shared unigenes in each population, and analyzing the relationship between Ed and π across populations.

To investigate the role of genetic and expression components in environmental adaptation, we compared genetic diversity and expression diversity between high- (northern, DL and QD) and low-latitude (southern, ZS and XM) populations, using Wilcoxon paired tests to assess the significance of differences in π and Ed.

### Identification of temperature-relevant candidate genes and candidate gene transcripts

We searched the Web of Science (https://webofscience.clarivate.cn/wos/) using various keyword combinations, including temperature, heat tolerance, thermal adaptation, etc., focused on studies that experimentally confirmed the relevance of genetic loci to temperature in arthropods. These genes were considered to be subject to natural selection pressure mediated by temperature. We then identified temperature-relevant reference candidate genes based on these studies. The number of temperature-relevant genes obtained from each database included 11,608 from FlyBase, 3,008 from UniProt, and 2,366 from NCBI (Table S1). These genes were then BLAST compared against the filtered unigenes from the population transcriptome of *O. oratoria* in this study. The comparison results were screened based on the following parameters: e-value < 1e-3, identity > 50%, and sequence length of the target gene > 50% of the matched sequence. Transcripts that matched the reference candidate genes were classified as ‘Candidate Gene Transcript’ (CGT). We identified the non-CGT sets by excluding the CGT sequences from the reference *O. oratoria* SMRT full-length transcriptome. PCA was performed based on the SNP and expression data of CGTs, respectively. Dispersion coefficients derived from FPKM values were used to estimate the expression variability of genes expressed in more than 50% of the samples. Subsequently, a two-sample t-test was conducted to compare variability between CGT and non-CGT sets, with log transformation applied to ensure normality.

### Identification and functional statistics of differentially expressed and highly divergent transcripts in northern and southern group

DESeq2 was used to identify differentially expressed transcripts between two groups (Northern group: DL & QD, Southern group: ZS & XM), considering significance at adjusted *p*-values < 0.05 and fold changes > 1.5. Up- and down-regulated transcripts across pairwise contrast were categorized as ‘Differentially Expressed (DE)’ transcripts between northern and southern group.

The discriminant analysis of principal component (DAPC) analysis performed in the R package Adegenet v1.3.1 [36] included pairwise comparison to assess allele loadings and identify bi-allelic SNPs driving group divergence, covering more than 99% of the variance. Following the method described by Herrmann et al*.* [21], a 10% threshold for allele loadings was applied to identify the top 10% of SNPs that significantly contributed to genetic differentiation between groups, which were labelled ‘highly divergent’ and transcripts containing these SNPs were labelled ‘Highly Divergent (HD)’ transcripts between different groups.

Gene ontology (GO) terms were obtained from UniProt (http://www.uniprot.org/) for all highly divergent and differentially expressed CGTs. The functional overlap between these transcript types was analyzed by assigning each transcript to its deepest non-overlapping GO term and comparing the proportions of shared GO terms with the proportions of shared transcripts using z-tests.

### Proportional difference and over-representation analyses

Given the significant impact of temperature on genetic divergence among *O. oratoria* populations [19], the influence of temperature on expression divergence was further considered. We assessed temperature-relevant genetic divergence in *O. oratoria* populations by calculating the proportions of differentially expressed and highly divergent CGTs relative to the total group-specific transcripts. In addition, hypergeometric tests were used to test for over-representation of CGTs in the results of the DAPC and differentially expressed analyses relative to the set of input transcripts for each analysis (R function phyper, R Core Team 2014), with Bonferroni correction applied for multiple comparisons.

## Results

### Characteristics of transcriptome

Illumina sequencing of 51 *O. oratoria* individuals generated approximately 2.19 × 10^9^ raw reads (329.02 Gb), with an average of 4.29 × 10^7^ raw reads (6.57 Gb) per sample (Table S2). After quality control, approximately 2.15 × 10^9^ clean reads were retained, with an average Q20 of 97.57% (Table S2). The clean reads were mapped to the *O. oratoria* reference sequences, detecting 88.47%−93.70% of the reference transcriptome in each individual (Table S2), indicating high sequencing quality for further analysis.

### Population structure

PCA and correlation analysis of the transcriptomic data from 14,291 transcripts indicated that individuals clustered into northern (high-latitude) and southern (low-latitude) groups according to their geographic locations (Fig. 1b, Fig. S1). This was supported by the results of PCA and STRUCTURE analysis using SNP data from *O. oratoria* populations (Fig. 1c and d), with the optimal K-value detected by the CV error plot being K = 2 (Fig. 1e). In addition, pairwise *F*_ST_ values between *O. oratoria* groups ranged from 0.65 to 0.66 (*p* < 0.05, Table S3), indicating significant genetic differentiation (*F*_ST_ > 0.15 is generally considered significant) [37].

### Patterns of population expression and genetic diversity

**Fig. 2:**
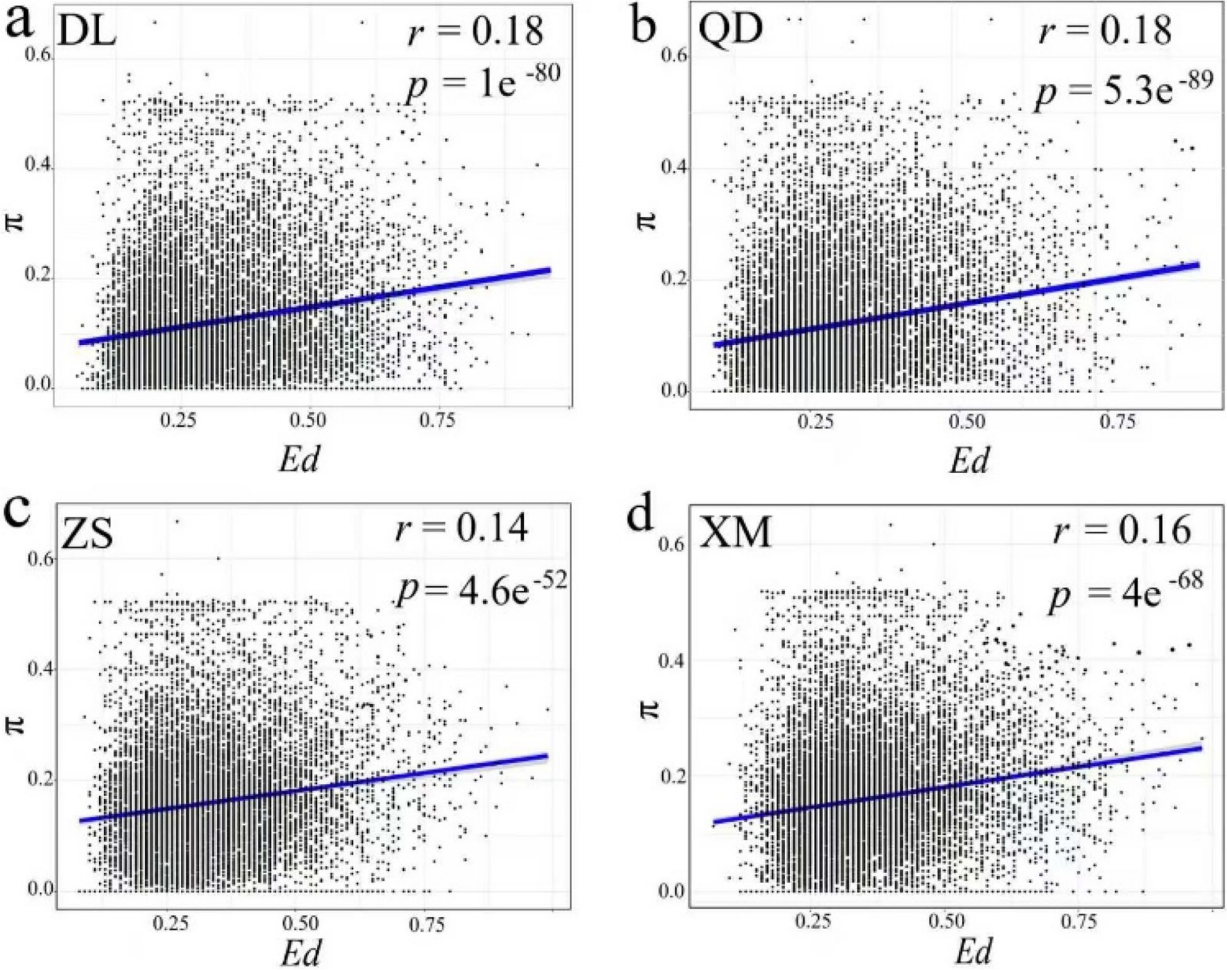
Correlation between the genetic and expression diversity of all transcripts within four *O. oratoria* populations. **a** DL population. **b** QD population. **c** ZS population. **d** XM population

Within each population, a significant positive correlation between Ed and π at the gene level was detected, with values ranging from *r* = 0.14 to 0.18 (*p* ≤ 4.6e-52) across all *O. oratoria* populations (Fig. 2).

**Fig. 3:**
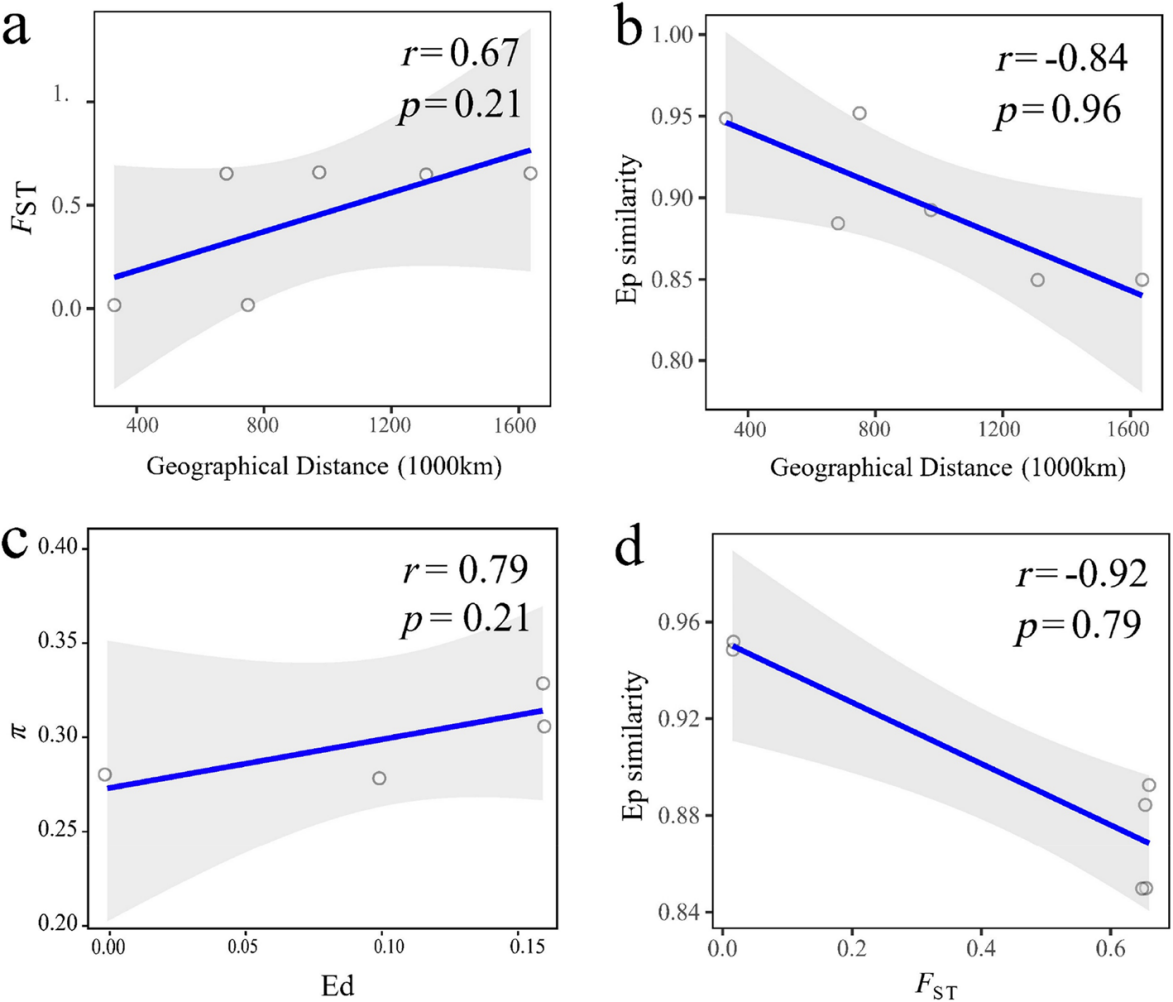
Relationships among geographic distance, genetic differentiation, and expression similarity across *O. oratoria* populations. **a** Correlation between geographic distance and genetic differentiation. **b** Correlation between geographic distance and expression similarity. **c** Correlation between the genetic diversity and expression diversity of all transcripts among 4 populations. **d** Correlation between genetic differentiation and expression similarity

Between populations, the Mantel tests indicated that no significant relationship was detected between pairwise *F*_ST_ and geographic distance (*r* = 0.67,* p* = 0.21, Fig. 3a), as well as between Ep similarity and geographic distance (*r* = − 0.84, *p* = 0.96, Fig. 3b), suggesting the absence of isolation by distance (IBD). Furthermore, expression diversity and genetic diversity across populations showed a non-significant positive correlation (*r* = 0.79, *p* = 0.21) (Fig. 3c). Consistent with this, gene expression (Ep) similarity and genetic differentiation across populations exhibited a non-significant negative correlation (*r* = − 0.92, *p* = 0.79) (Fig. 3d). When considering different groups, the genetic diversity of southern group was significantly higher (Wilcoxon paired test, *p* < 0.01) than that of the northern group (Fig. S2a). This pattern was mirrored in expression diversity, with the southern group also showing significantly higher values (Wilcoxon paired test, *p* < 0.01) (Fig. S2b).

### Temperature-adapted candidate gene transcripts, differentially expressed and highly divergent transcripts

We obtained 38,254 reference candidate genes associated with thermal adaptation in arthropods from 50 studies on thermal adaptation (Table S1). Of these, 2,865 *O. oratoria* transcripts were mapped to 16,982 reference candidate genes and categorized as CGTs. PCA analysis based on CGTs data showed that there was a highly interpreted pattern of north–south genetic differentiation in *O. oratoria* populations (Fig. S3). In addition, dispersion quartiles calculated for 2,322 CGTs and 13,387 non-CGTs showed significant mean differences (0.47 vs. 0.40; two-sample t-test, df = 15,705, *p* < 0.001) despite a small disparity of 0.07 (Table S4).

**Fig. 4:**
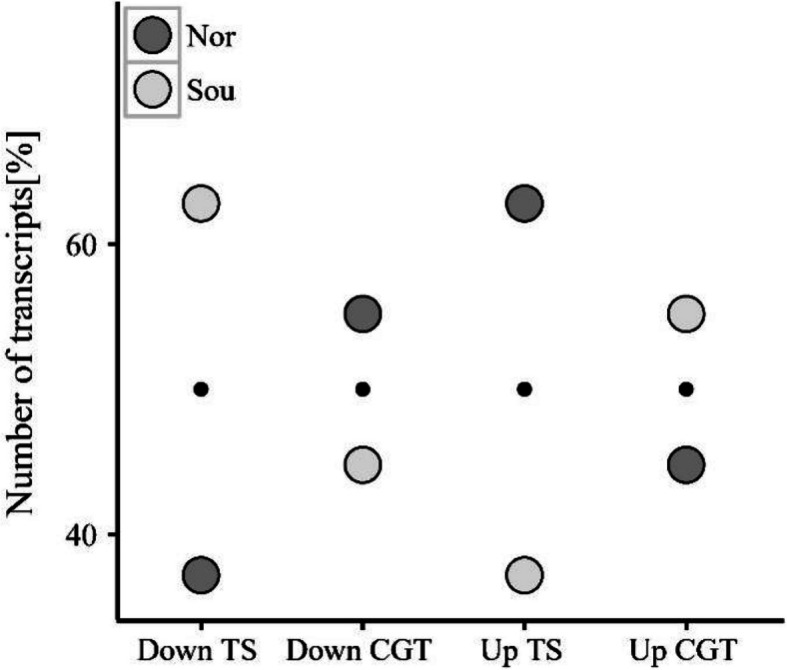
Proportions of differentially expressed transcripts in each population (relative to total transcripts analyzed). TS, all transcripts. CGT, candidate gene transcripts. Down, differentially downregulated transcripts. Up, differentially upregulated transcripts

From pairwise DAPC analyses between northern and southern group, we identified SNPs that significantly contributed to sequence divergence. The number of ‘highly divergent’ SNPs is 8,456 in comparisons, corresponding to 482 transcripts (Table 1 & Table S5). In addition, we further identified 6,624 ‘differentially expressed’ transcripts and 475 ‘differentially expressed’ CGTs between different groups (Table 1 & Table S5). We observed that the proportions of differentially expressed transcripts varied between the full transcript set and the CGT subset (Table 2, Fig. 4). Notably, the proportion of differentially downregulated CGTs was higher than that of downregulated transcripts in the northern group, whereas differentially upregulated transcripts followed this pattern in the southern group (Fig. 4).

**Table 1:** Transcript and SNP datasets in various analyses; and detected differentially expressed and highly divergent transcripts

	**Analyzed transcripts (SNPs)**	**Analyzed CGTs (SNPs)**	**# Detected transcripts (SNPs)**	**# Detected CGTs (SNPs)**
HD total	16,458 (264,355)	1,040 (11,020)	8,456 (26,436)	482 (1,130)
DE	14,291	905	6,624	475
DE Nor up			4,162	213
DE Sou up			2,462	262
DE Nor down			2,462	262
DE Sou down			4,162	213

Analyses of differential expression were conducted at the transcript sequence level. In DAPCs, SNPs corresponding to a specific number of transcripts were analyzed to identify genetic variations accounting for population divergence

*HD* Highly divergent transcripts according to DAPC (the same input was analyzed in principal component analysis), *DE* Differentially expressed transcripts, *CGT* Candidate gene transcript

^#^Detected transcripts/CGTs: number of differentially expressed or highly divergent transcripts or CGTs

**Table 2:** Hypergeometric tests for over-representation of candidate gene transcripts in test results

Analysis	CGT input [%]	CGT output [%]	Difference [%]
DAPC	6.3	5.5	0.8
DE Nor up		5.1	1.2
DE Sou up		10.6	4.3^**^
DE Nor down		10.6	4.3^**^
DE Sou down		5.1	1.2

Significant results are marked in bold

^**^p <.01

### Over-representation analyses

Hypergeometric tests were performed to assess the over-representation and significance of candidate gene transcripts in the results of the DAPC and differentially expressed analyses. The results indicated non-significant findings for higher proportions of CGTs among highly divergent transcripts compared to the input datasets (Table 2). Notably, significant over-representation of CGTs was found in differentially up- and down-regulated transcripts, with the southern and northern group showing 4.3% more CGTs than expected (*p* < 0.01), respectively.

### Functional consistency of differentially-expressed and highly divergent CGTs

A significant proportion of gene functions are found to be shared between subsets of highly divergent CGTs and differentially expressed CGTs. Specifically, 51.5% of biological process (BP) GO terms associated with highly divergent CGTs were found to overlap with differentially expressed CGTs, while 37.8% of differentially expressed CGTs’ BP GO terms overlapped with highly divergent CGTs. A similar pattern was observed in molecular function (MF) GO terms, where 52.2% of MF GO terms annotated in highly divergent CGTs overlapped with differentially expressed CGTs, and 76.6% of differentially expressed CGTs’ MF GO terms overlapped with highly divergent CGTs (Table 3). Proportionally, there are significantly more shared BP (*p* < 0.05, z-tests) and MF GO terms (*p* < 0.01, z-tests) between differentially expressed and highly divergent CGTs compared to transcripts shared between these two types of CGTs (Table 3). A total of 56 GO terms were identified as common functions among highly divergent and differentially expressed CGTs in *O. oratoria* populations, primarily related to cellular metabolism, signaling, and regulation of gene expression (Table S6).

**Table 3:** Summary of GO terms associated with highly divergent and differentially expressed candidate gene transcripts

GO category and analysis		# CGT	GO term	GO term/TS	CGTs shared with		GO terms shared with		Significance of differences in response overlap (CGT and GO)
					**DAPC (%)**	**DE (%)**	**DAPC (%)**	**DE (%)**	
BP	DAPC	317	33	0.24	/	24.3	/	51.5	** (*p* < 0.05, z-test)
	DE	212	45	0.47	36.3	/	37.8	/	
MF	DAPC	340	69	0.29	/	25.0	/	52.2	** (*p* < 0.01, z-test)
	DE	317	47	0.21 254.5	26.8	/	76.6	/	
CC	DAPC	58	17	0.41	/	27.6	/	17.6	Ns (*p* = 0.67, z-test)
	DE	61	13	0.26	26.2	/	23.1	/	

The table contains only CGTs associated with GO terms

*GO* Gene ontology, *BP* Biological process, *MF* Molecular function, *CC* Cell component

^#^CGT: number of candidate gene transcripts (with associated GO terms)

## Discussion

### The synergetic effect of genetic and expressive diversity in *O. oratoria* adaptation

Adaptations can occur along continuous environmental gradients, even in the absence of geographic isolation, which are generally attributed to nature selection [38, 39]. In this study, phylogeographic analyses based on transcriptome-wide SNP and gene expression data reveal two highly-diverged lineages in *O. oratoria* with low within-lineage and high inter-lineage genetic differentiation, corroborating previously reported geographic patterns of genetic differentiation in line with the NWP biogeographic boundary around the Yangtze River Estuary [17–19]. This spatial genetic structuring in *O. oratoria* arises through restricted gene flow [17]. Dispersal restriction and geographic isolation typically interact to drive adaptive differentiation via selection-mediated fitness tradeoffs [31]. However, we found no IBD signal at the kilometer scale, as neither genetic divergence nor expression similarity exhibited significant correlations with geographic distance (Fig. 3a, b). These findings collectively implicate environmental filtering as the predominant driver shaping both genetic and expression divergence across populations in this widely distributed mantis shrimp. In fact, historical isolation patterns, along with contemporary biotic and abiotic factors—such as oceanic temperature-salinity gradients, ocean currents, and species life history traits, jointly shape spatial patterns of adaptive differentiation in marine organisms [40]. Notably, palaeohydrological events and contemporary latitudinal spans and ocean current systems have significantly influenced the spatially heterogeneous temperature selection pressures on the mantis shrimp *O. oratoria* along latitudinal gradients [19], which are also key environmental drivers of genetic variation in other marine organisms in the NWP [41, 42].

It has generally been recognized that gene expression diversity serves as a pivotal strategy of adaptation in wild populations inhabiting heterogeneous environments (e.g., [32, 33]). Despite varying degrees of environmental stress experienced by different *O. oratoria* populations, we detected a significant and consistent positive correlation between genetic diversity and gene expression diversity at the gene level within four populations (Fig. 2). This suggests a reciprocal relationship between the expression and nucleotide diversity of genes when adapting to the organism’ s surroundings. Such a pattern likely results from genetic regulation, where changes in regulatory elements affect expression levels [43]. In addition, certain genomic regions may exhibit increased nucleotide and expression diversity due to their crucial functions [44], possibly implying the existence of functionally important genomic regions critical for environmental adaptation in *O. oratoria*. Notably, the persistence of this covariance of nucleotide and expression diversity in all observed *O. oratoria* populations implies its heritable nature, potentially conferring survival advantages through enhanced individual fitness [45, 46].

Interestingly, nucleotide diversity was also found positively correlated with gene expression diversity among populations (Fig. 3c), and both the nucleotide diversity and expression diversity of the overall population were elevated at southern latitudes relative to northern latitudes. A major issue when interpreting genetic diversity is to account for historical demographic processes that can profoundly shape contemporary genetic diversity. The demographic history of *O. oratoria* has been subject to repeated population contraction and expansion cycles driven by Quaternary glacial-interglacial cycles [17, 19]. Strong demographic fluctuations have been detected in both northern and southern ancestral populations of *O. oratoria* [17, 19]. Furthermore, contemporary distributions of northern and southern lineages are assumed to result from postglacial recolonization originating from two major glacial refugia in the NWP: the Japan Sea and the South China Sea [17]. Unlike the more thermally variable Japan Sea, the South China have historically experienced prolonged periods of stable high temperature [47, 48], possibly allowing southern *O. oratoria* populations more evolutionary time to accumulate genetic diversity.

Collectively, all these results pinpoint the synergetic effect of genetic and expressive diversity on the adaptation of *O. oratoria* to the heterogenous seascape of the NWP and population persistence in diverse environments. However, our findings contrast with previous reports of non-relevant or negatively correlated relationship between nucleotide diversity and gene expression diversity at the gene level within populations [48, 49], indicating that this mechanism may not be universal and could be specific to particular lineages or habitats.

### The potential role of temperature selection in gene expression divergence

Given that the information on gene-phenotype relationships is scarce in *O. oratoria*, we employed a reverse ecology approach and focused on analyzing transcripts that are orthologous to candidate genes for arthropod thermal adaptation, which are considered to be subject to natural selection mediated by temperature stress. Our results revealed an over-representation of differentially up-regulated CGTs among up-regulated transcripts in southern populations (ZS and XM), implying the operation of local thermal selection on the expression level. These constitutive expression differences may have been shaped by thermal selection and represent early processes in adaptive divergence [50–53]. Furthermore, transcripts with higher expression variability were found to be more abundant in the CGT subset, thereby suggesting a considerable evolutionary potential in *O. oratoria* with respect to temperature [54]. Admittedly, we acknowledge that the transcriptional differences observed between the northern and southern geographic populations may not be exclusively due to natural selection; plastic responses to local climatic conditions experienced in the filed could also influence the analysis of wild-caught individuals. Nevertheless, several lines of evidence suggest that natural selection may have played a role in shaping expression divergence. Specifically, the lack of IBD in both genetic and expression divergence, coupled with strong latitudinal population structure, is consistent with adaptive differentiation. In addition, functional over-representation analyses revealed strong signals of temperature-related adaptation in candidate thermotolerance genes (CGTs), adding further support of the involvement of selective processes.

Notably, temperature-relevant CGTs were not over-represented among highly divergent transcripts, further indicating a stronger influence of thermal selection on the expression level. This parallels previous research that have emphasized the importance of expression variation over coding sequence variation in driving parallel phenotypic divergence in three-spined sticklebacks [55]. The absence of significant over-representation of CGTs at the coding sequence level, however, does not preclude the potential impact of temperature-mediated selection on sequence variation. This is because non-coding regulatory elements can influence gene expression without altering protein-coding sequences [56]. Extensive empirical studies, including those on the Atlantic Salmon and eastern oyster, have demonstrated that minor changes in allele frequency can influence responses to temperature fluctuations, possibly due to selection acting on linked regulatory regions or genes rather than coding ones [57–59]. In our study, CGTs showed differences between adaptive expression and sequence variation in some cases, such as Mitochondrial enolase superfamily member 1 (transcript/53392/f253p0/247 and transcript/54567/f2p0/2508), supporting the role of trans-regulatory factors in the decoupling between gene expression and coding sequence [60]. It needs to be emphasized that RNA-Seq may fail to capture intron and regulatory variation due to incomplete coverage [61–63], suggesting the need for genome resequencing to capture all relevant SNPs, especially in non-coding regions [64].

In addition to innate genetic factors, adaptation may also arise through phenotypic plasticity, or the combined action of both mechanisms [65]. From 2010 to 2020, the annual sea temperatures range experienced by *O. oratoria* at northern populations (DL: 24.21 °C; QD: 23.32 °C) considerably exceeded those at southern populations (ZS: 20.87 °C; XM: 14.26 °C) (https://bio-oracle.org) [66], suggesting that northern populations endure more variable thermal regimes. Previous studies on marine organisms have proposed that populations experiencing greater annual temperature fluctuations may exhibit higher phenotypic plasticity in thermal tolerance, which could affect the way genes are expressed differently in different populations [67, 68]. In this study, we found that, under natural conditions, southern populations exhibited greater expression diversity than northern populations. However, this finding does not preclude the possible influence of plasticity. Further comparisons of physiological responses of different latitude populations under the same thermal stress would provide insights into the importance of phenotypic plasticity on thermal adaptation of *O. oratoria* across a large-scale latitudinal gradient. Such insights will prove critical for predicting *O. oratoria* population resilience under ongoing climate warming.

### Functional convergence of sequence and expression variation

In the present study, we found that the subset of highly divergent CGTs is associated with a wider range of GO terms, encompassing biological processes, molecular functions, and cellular components compared to differentially expressed CGTs. The differences in GO term diversity and abundance between the two subsets suggest that the expression profiles of temperature-relevant CGTs under stress-free conditions are restricted to a narrow and specific set of biological functions, which may be due to different constraints on sequence and expression variation [69]. For example, factors such as genomic architecture have the potential to drive sequence divergence through local reductions in effective population size, while gene expression may be affected by pleiotropic effects where changes in regulatory elements affect multiple genes [70, 71]. In other words, divergent regulation at the expression level in *O. oratoria* is likely to be highly polygenic, suggesting that it may exhibit greater genetic redundancy than sequence variation determined by a single gene or molecular pathway, which exhibit limited repeatable genetic functions of adaptation [72].

Of particular note, 13.6% of the CGTs exhibit both distinctive expression levels and high levels of DNA sequence divergence, whereas 25% of their associated GO terms are shared across the two datasets. The differentially expressed and highly divergent CGTs exhibit greater overlap in functional categories, including the biological process and molecular function GO terms, compared to the gene set (Table 3). This implies that natural selection likely drive adaptation of *O. oratoria* populations to spatial temperature heterogeneity in the NWP by promoting functional alternative pathways for both genetic and regulatory variation, a mechanism similar to that documented in *Daphnia* [21], rather than through temperature-mediated natural selection acting simultaneously on gene sequence and expression levels. This supports the hypothesis that commonalities between genetic and regulatory variation may be observed at higher functional classifications but may be rare at the level of individual loci [73].

Despite the presence of notable discrepancies in absolute abundances, a certain degree of similarity in GO terms was observed between the two datasets. Enrichment analysis revealed that highly divergent and differentially-expressed CGTs together constitute the mitochondrial matrix cellular component. Key to biological thermal adaptation are mitochondria and their ability to provide metabolic energy. It has been reported that Antarctic fish have a lower metabolic range compared to more active temperate and tropical fish [74]. The mitochondrial matrix is the center of cellular energy metabolism, which is essential for maintaining cellular energy balance and life activities [75]. Consequently, differences in mitochondrial components may play an important role in the temperature-adaptive differentiation of *O. oratoria*. Furthermore, multiple shared GO entries related to signal transduction were found, specifically involving processes such as GTP binding, nucleotide binding and nucleoside phosphate binding. These findings suggest that cellular processes of sensing and responding to external signals may play an important role in enabling *O. oratoria* populations to adapt to thermally heterogeneous environmen [76].

## Conclusion

In this study, we demonstrated that genetic and expression variation played crucial roles in the adaptive divergence of *O. oratoria* populations distributed along a latitudinal gradient in the NWP, collectively influencing the capacity of *O. oratoria* to adapt to environmental heterogeneity. Differentially expressed temperature-adapted candidate transcripts were significantly over-expressed, suggesting tha variability in expression may play a key role in mediating responses to thermally environmental stress. Conversely, highly differentiated transcripts did not show significant over-expression, emphasizing the necessity for further exploring the potential regulatory role of non-coding regions. The expression-specific and highly differentiated candidate gene transcripts showed functional consistency and may constitute functional alternative pathways in response to thermally environmental stresses across different latitude populations. Our work highlights the imperative to integrate genetic and expression variation to improve our understanding of species adaptation and evolutionary processes in the face of climate change, thereby offering novel perspectives on adaptive strategies within marine organisms.

## Supplementary Information

Supplementary Material 1: Table S1. Temperature-relevant reference candidate genes in arthropods from studies confirmed experimentally

Supplementary Material 2: Table S2. Sequencing data and mapping rates of 51 individuals based on the reference transcriptome

Supplementary Material 3: Table S3. Pairwise *F*_ST_ comparisons between populations inferred from the high-quality SNP data sets.

Supplementary Material 4: Table S4. Dispersion coefficients for expression in all individuals for both CGT and non-CGT data sets

Supplementary Material 5: Table S5. Highly divergent or differentially expressed CGTs and functional annotations between northern and southern populations

Supplementary Material 6: Table S6. Common GO terms of highly divergent and distinctively expressed CGTs in* O. oratoria* populations.

Supplementary Material 7.

## Data Availability

Illumina sequencing data generated by this study have been submitted to NCBI Sequence Read Archive (SRA) under the BioProject accession number PRJNA1282866.

## Abbreviations

NWP: Northwestern Pacific
CGTs: Candidate gene transcripts
DL: Dalian
QD: Qingdao
ZS: Zhoushan
XM: Xiamen
MAF: Minor allele frequency
*π*: Nucleotide diversity
*F*_ST_: Pairwise genetic differentiation
PCA: Principal component analysis
FPKM: Fragments per kilobase of transcript per million fragments
Ep: Gene expression
Ed: Gene expression diversity
*r*: Pearson correlation coefficients
DE: Differentially expressed
DAPC: Discriminant analysis of principal component
HD: Highly divergent
GO: Gene ontology
SST: Sea surface temperature
IBD: Isolation by distance
SCSWC: South China Sea Warm Current
TWC: Taiwan Warm Current
CCC: China Coastal Current
CRDW: Changjiang River Diluted Water
YSWC: Yellow Sea Warm Current
TSWC: Tsushima Warm Current
LC: Lima Current
BP: Biological process
MF: Molecular function
CC: Cell component
