# Supplementary material for: Concerted genetic and transcriptomic shifts underlie adaptation to a latitudinal thermal gradient within a widespread mantis shrimp
Source: BMC Ecol Evol. 2025 Oct 17;25:109. doi: 10.1186/s12862-025-02452-1 (PMC12535113; doi:10.1186/s12862-025-02452-1)
Supplement: Supplementary file 7 — Supplementary Material 7. [file 12862_2025_2452_MOESM7_ESM.docx]

Supplemental information for

**Population transcriptomics reveals adaptation to a latitudinal thermal gradient within a widespread mantis shrimp**

**Supplementary data**

**Table S1.xls**

**Table S1.** Temperature-relevant reference candidate genes in arthropods from studies confirmed experimentally.

**Table S2.xls**

**Table S2.** Sequencing data and mapping rates of 51 individuals based on the reference transcriptome.

**Table S3.xls**

**Table S3.** Pairwise *F*_ST_ comparisons between populations inferred from the high-quality SNP data sets.

**Table S4.xls**

**Table S4.** Dispersion coefficients for expression in all individuals for both CGT and non-CGT data sets.

**Table S5.xls**

**Table S5.** Highly divergent or differentially expressed CGTs and functional annotations between northern and southern populations.

**Table S6.xls**

**Table S6.** Common GO terms of highly divergent and distinctively expressed CGTs in *O. oratoria* populations.

**Figure S1.jpg & Figure S1.pdf**

**Figure S1.** Heatmap of expression-based correlations between 51 samples.

**Figure S2.jpg & Figure S2.pdf**

**Figure S2.** Genetic diversity (a) and expression plasticity (b) in northern and southern groups. Significant results (Wilcoxon paired test) are presented.

**Figure S3.jpg & Figure S3.pdf**

**Figure S3.** PCA analysis based on CGTs data.(a) Population genetic of *O. oratoria* using PCA analysis based on FPKMs of identified CGTs. (b) PCA of 51 individuals based on SNPs of identified CGTs.


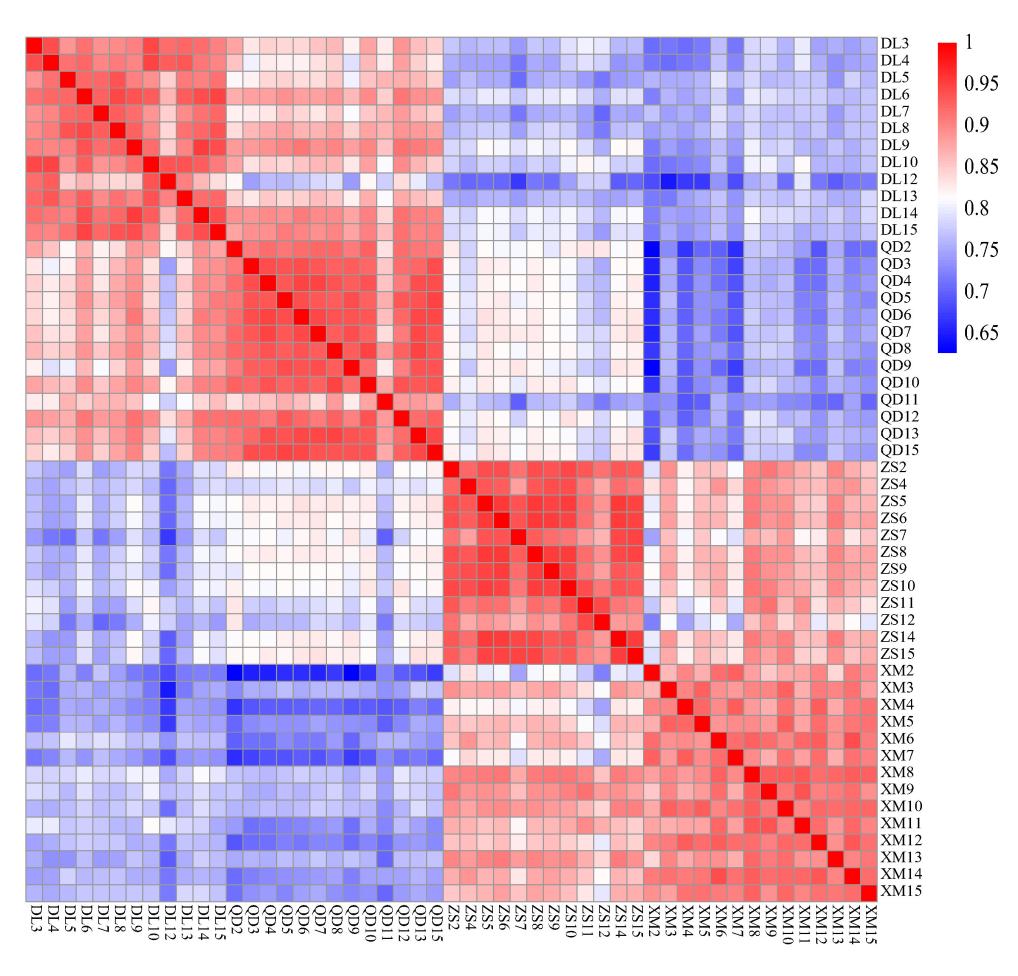


**Figure S1** Heatmap of expression-based correlations between 51 samples.


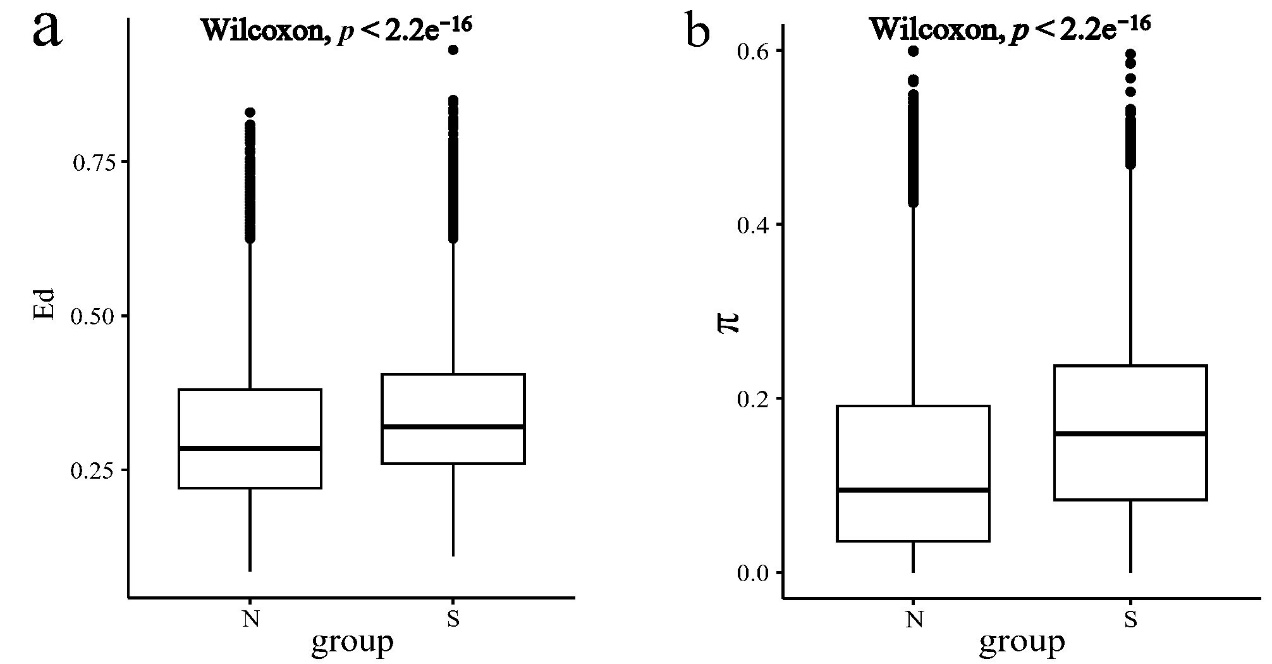


**Figure S2** Genetic diversity (a) and expression plasticity (b) in northern and southern groups. Significant results (Wilcoxon paired test) are presented.


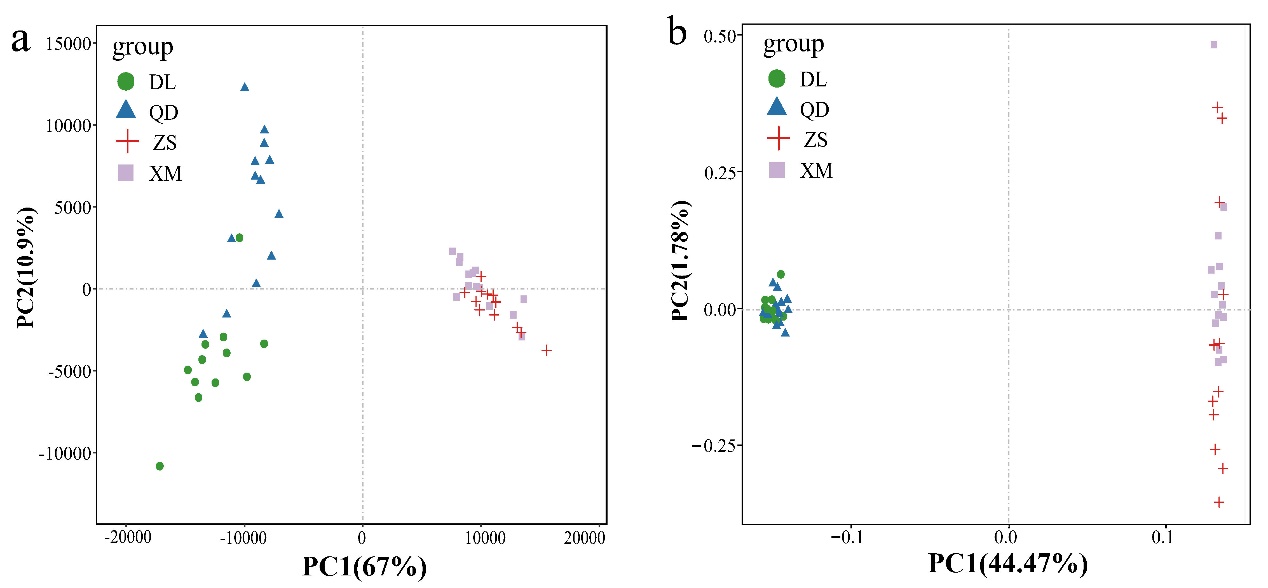
**Figure S3** PCA analysis based on CGTs data. (a) Population genetic analyses of *O. oratoria* using PCA analysis based on FPKMs of identified CGTs. (b) PCA analysis of 51 individuals based on SNPs of identified CGTs.
